# The Connectedness of Packed Circles and Spheres with Application to Conductive Cellular Materials

**DOI:** 10.1371/journal.pone.0051695

**Published:** 2012-12-20

**Authors:** John P. Swensen, Aaron M. Dollar

**Affiliations:** 1 Mechanical Engineering and Materials Science, School of Engineering and Applied Science, Yale University, New Haven, Connecticut, United States of America; King's College London, United Kingdom

## Abstract

In this paper, we examine the static connectivity of 2D and 3D arrays of spherical cells with conductive paths, and the associated power dissipation in the individual cells. Herein, we use the term “cellular material” to describe the ensemble of many cells, in contrast to the more traditional use of the term for foams and honeycomb materials. Using a numerical analytical approach from highly parallel resistor arrays, we examine the cells and ensemble structures in terms of their connectivity, defined as the number of cells that are dissipating power, as well as the redundancy and robustness to localized cell failure. We examine how the connectivity changes with the geometry of the conductive cell surface area, and in particular, the percentage of the cell half that is conductive and makes contact with neighboring cells. We find that the best connectivity exists when the conductive surface of the cell is approximately 80% of the hemisphere surface, addressing the tradeoff of maximizing contact with neighboring cells while minimizing shorts in the structure. In terms of robustness, the results show that, for the proposed circular and spherical cell design, the connectivity is a nearly linear function of the number of disconnects, indicating that there is not a catastrophic effect of isolated cell failures. In terms of structure size, the connectivity appears to plateau at around 60% for the planar structures and around 50% for the cubic structures of around 500 cells or greater with random cell orientation.

## Introduction

**Figure 1:**
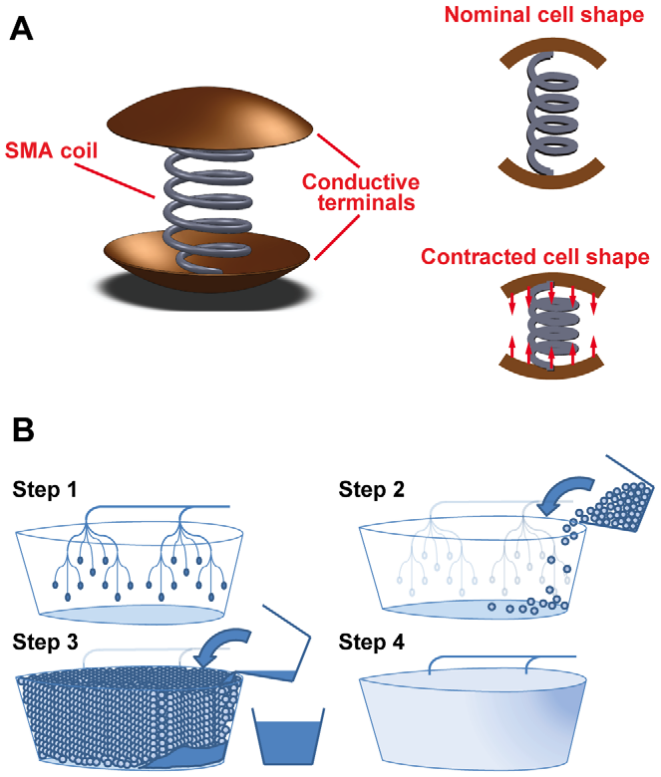
Proposed cell design and fabrication method. A. A potential cell design with a contractile shape memory alloy (SMA) component and conducting terminals. Current flowing through the SMA coil, due to an electrical potential between the two terminals, causes resistive heating and subsequent cell contraction, and B. A potential fabrication method of packing many cells into a mold or arbitrary shape and adhering the cells together with a binding agent. The properties of the structure can be adjusted through cell design and the material properties of the binding agent.

This paper presents work related to the analysis and design of spherical cells that, due to conductive contact with their neighbors, form spatial conductive arrays. This type of system has potential applications in a number of different areas, such as active materials [1]–[3], multifunctional and smart structures [4], [5], or basic science related to packing [6], [7]. The authors, for instance, are interested in the eventual development of actuated cellular materials, where an active material actuator can form the core of a quasi-passive cell that contracts when current is passed through it. Large groups of these cells can be fused together in essentially arbitrary geometries to form complex articulated structures that would be difficult or impossible to make with traditional approaches (see Figure 1). From an engineering perspective, this approach may provide advantages over traditional mechanism and structures-based design, namely by providing a material basis for larger components, which can be produced inexpensively in high volume. This approach may also provide advantages to in constructing ensembles of cellular materials as flexible conductive elements or as sensor networks embedded in materials.

The focus of this paper is science precursory to the construction of the physical cells mentioned previously, namely an analysis of the connectivity and not the potential applications, so the remainder of the paper will focus on the connectivity under the following assumption: given cells with two-terminal surface geometry and connecting resistance, what parameters of the cell construction are relevant to determine the overall resistor network structure. The approaches taken in this paper draw on previous results of analyzing arbitrary resistive circuits as weighted graphs. An introduction to these techniques can be found in a review of distributed sensor network methods [8]. Other previous work analyzes the problem of solving arbitrary nodal network representations of resistive networks through more traditional circuit analysis techniques [9]–[11].

There exists a vast body of literature addressing the problem of jammed packings [12]–[15]. There also exists a significant amount of work on thermal conductivity in jammed packings. If we were considering the problem of electrical conductivity of solid or shell metallic sphere granular material, electrical conductivity would be quite related to the problem of thermal conductivity through the Wiedemann-Franz law [16]–[18]. However, the problem we are addressing is different in two key aspects due to the construction of the cells presented in this paper: (1) the proposed cell design has two conducting terminals that do not span the entire hemi-circle/sphere and (2) the conductive element connecting the two terminals through the center of the cell is a different material than the conducting terminals.

## Methods

### A. Cell design

The most important aspect of the cell design is the geometry of the cell terminals and material connecting the two terminals. Each cell consists of two conducting terminals with a conductive element connecting the two terminals, as shown in Figure 1. Because the cell terminals do not span the entire hemi-circle/sphere of the cells, the connectivity is different than that of conductive granular material. The size of the terminals is one of the primary variable parameters explored in this paper and the effect on connectivity as a function of the cell terminal size and the random orientation of the cells in the packing.

### B. Cell packing

**Figure 2:**
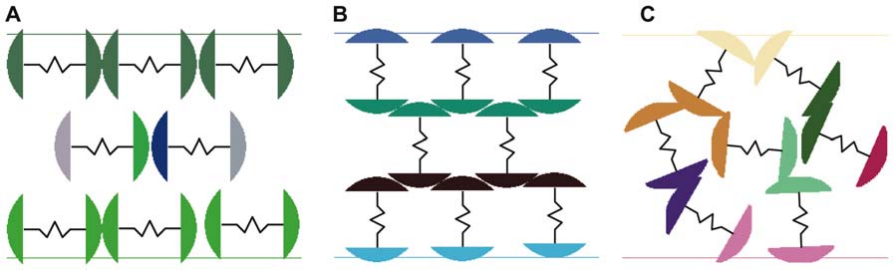
The effect of cell alignment on top to bottom connectivity. The type of cell packing and cell orientation play a critical role in the parallel-series electrical connectivity between two points in the cell arrangement. Here we show the worst, best, and typical connectivity: A. a face-centered cubic cell arrangement that results in no connectivity between rows of cell terminals, where no current will flows through the cells, B. an ideal cell arrangement in a face-centered cubic packing and vertical alignment, which results in maximal parallel and series connectivity, and C. a typical cell arrangement with random packing and random orientation, where the degree of parallel and series connectivity is between the pathologic and ideal cases.

After determining the geometry of the active cells – herein taken to be nominally circular 2-dimensional cells and spherical 3-dimensional cells – the next aspect of critical importance is the packing of the cells. While we take a basic approach to cell packing in this paper, many of the techniques, terminology, and considerations for analysis existent in the packing literature will be applicable for any packings that result during the process of arranging cells. A thorough review of jammed hard-particle packing, including the effects of geometry, achievable configurations, and the lexicon for packing literature are given by Torquato and Stillinger [13]. Other packing literature also takes into account inter-particle interactions such as friction, normal forces, and gravity [7], [19]. In the simulations given in this paper, we modify the packing technique from [20] to consider cells that are first randomly placed, then packed by moving the upper boundary until jammed packing is achieved. During these simulations, inter-cell forces are computed and arbitrary viscous damping of cell motion is applied for packing stability. A small-scale example of indicative packing is shown in Figure 2-C. Figure 2-A and 2-B show worst-case and best-case examples of packing, respectively.

From a macroscopic view of the entire structure, the packed structure begins to look like a bulk conductor, where the degree of homogeneity is related to the degree of cell connectivity. When able to treat the structure as a homogenous bulk conductor, the equivalent resistance across the structure is
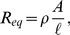
(1)where 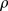, 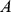, and 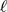 are the characteristic parameters for the resistivity, cross-sectional area, and length of the cell materials, respectively. Note that 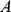 is not the cross-sectional area of the structure because a relatively small portion of the structure, namely the cell core and cell terminals, is conductive. A characteristic cross sectional area could be determined by an analysis of each prospective cell design.

Another characteristic that plays a role in determining this characteristic bulk conductance is the cell-to-cell contact conductance through the ensemble. The contact conductance is a function of the material properties of the cell terminals, the normal force between adjacent cells, and the radius of the cells [21]. In our analysis, we assume the simulations of jammed packings are executed such that they result in cell-to-cell forces that are approximately equivalent. The relationship between contact resistance and contacting area is
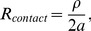
(2)where 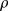 is the conductance of the terminal material and 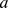 is the contact surface area between adjacent cell terminals. In turn, the contact surface area between adjacent cell terminals is
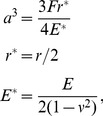
(3)where 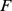 is the normal force between cells, 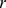 is the radius of each cell, 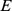 is the elastic modulus of the cell terminal material, and 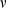 is the Poisson ratio of the cell terminal material. Given jammed packings with uniform intercellular forces, the contact resistance between each cell terminal pair will also be uniform. Interestingly, given non-zero contact resistance between touching cell terminals, the topology of the parallel-series resistive network does not change. However, changing contact resistance will impact how much power and where power is dissipated within the ensemble structure. To fully analyze the impact of contact resistance, the ratio of contact resistance, 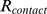, and cell core resistance, 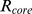, can be examined. From a microscopic view of small regions of the structure, the size and shape of the entire structure will define the boundary conditions. Current concentration near the boundaries, similar to temperature concentrations in problems of heat conduction and stress concentrations in problems of solid mechanics, will necessarily occur in our array of active cells. To help alleviate these current concentrations near the boundaries, which could result in irreparable damage to cells or excessive heating, we connect the current source across a large number of cells at opposite boundaries of the structure.

### C. Ensemble connectivity

**Figure 3:**
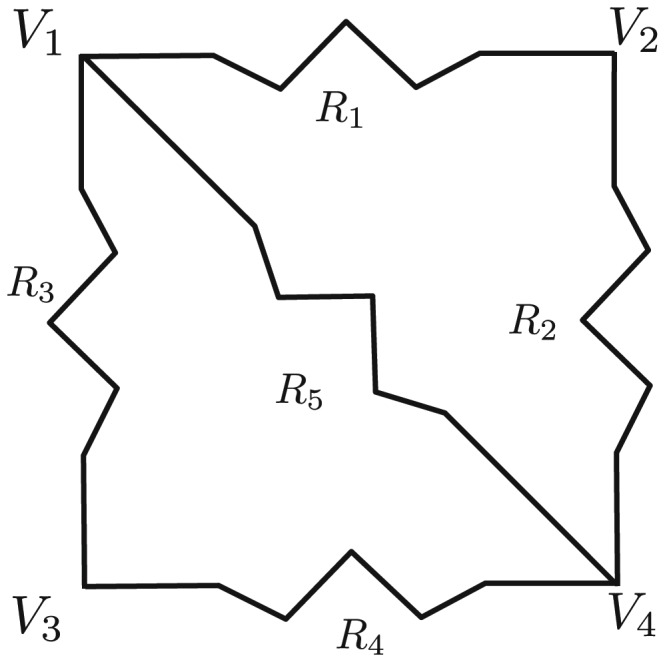
An illustrative parallel-series resistor network analyzed via Kirchhoff's laws.

Here, we analyze the connectivity of the structure by finding the equivalent parallel-series resistor network and solve the Kirchhoff's nodal current equations. This analysis allows us to specify 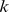 node currents and 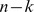 node voltages and solve for the remaining 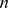 unknowns. However, for the sake of simplicity, throughout the rest of this paper we will assume that there is a current source connected to two terminals that span two entire edges of cell array. To illustrate the method, consider the simple parallel-series resistor network shown in Figure 3. The node current equations for this example are
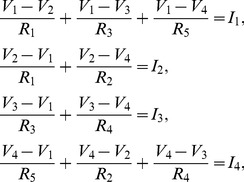
(4)where 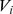 are the node voltages, 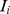 are the node currents, and 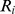 are the individual resistor values. We manipulate Equation (4) by writing each resistance as its equivalent conductance, 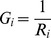, and writing the node currents as a linear function of the node voltages,
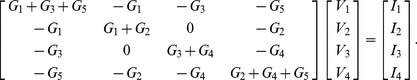
(5)


More generically, given an arbitrary resistor network represented as a graph with vertices being the nodes of equivalent electrical potential and edges having the equivalent resistance between nodes, the problem can be written as
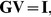
(6)where 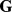 is the Laplacian relating node voltages and node currents. By nature, the Laplacian 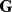 is necessarily a rank deficient matrix. This implies that as long as the vector of node currents is in the column span of 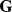, then solving for the node voltages will be unique up to a constant bias factor. The eigenvectors of 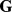 are such that any vector of node currents where
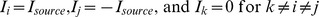
is also in the column span of 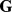. Because we are considering the power dissipated through Joule heating in each cell, this bias factor will be irrelevant,
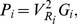
(7)where 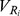 is the voltage difference across the *i*-th resistor, 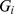 is the conductance of the *i*-th resistor, and 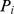 is the power in Watts dissipated in the same resistor. The exact value of 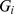 will be a function of both the cell's internal resistance and the contact resistance between adjacent cells. For a static structure where the cell-to-cell forces are equivalent, the contact resistance does not change the topology of the resistive cell network. However, it will change how much power is being dissipated in the cell's internal resistance vs. how much power is being dissipated in the cell terminals.

Though not explored in this paper, one advantage of this analysis technique it that is provides a natural method for further describing the network of cells as a time-varying system, where the resistance/admittance, power dissipation, and connectivity change as a function of the independent variable time. The method of analyzing the circuit through Kirchhoff's laws for node voltages and node currents trivially extends to structures with three-dimensional connectivity. At no point was it necessary to resort to Y-Δ transforms to simplify and analyze the resistive array.

## Results

We make several simplifying assumptions during the simulations presented herein. First, the simulations represent the power dissipated in each cell. This does not necessarily indicate the actual contraction of each cell as the relationship between power dissipation, temperature increase, and eventual contraction depends significantly on properties of the ensemble structure. In particular, thermal conductance properties of the cells and binding material will affect the relationship between power dissipation and temperature. The mechanical properties of the binding material will affect actual displacements of the structure because the structure shape will always be at an energy minimum of the store elastic energy between the cells and the binding material. Finally, we consider the effects of temporary cell separations that may occur during contractions of large numbers of cells. Certain levels of stress concentration in local regions of the connective substrate may cause temporary terminal separation during contraction of the active cells.

### A. Cell packing

In all simulations, cells are packed together using a simulation of elastic circular/spherical particles with viscous damping as described in [20]. Each cell is assumed to be an elastic particle with coefficients of stiffness and damping. Three and seven sides of the rectangular bounding box are fixed, for the 2D and 3D cases respectively. The upper boundary is slowly moved downward until jammed packing is achieved with an average interparticle force above a specified threshold. In all cases, the final packing fraction was greater than 78% for 2D packings and greater than 52% for 3D packings. The orientation of each cell was randomly chosen to determine the effects of the cell terminal sizes and the number of cells in the packing on the connectivity and power dissipated. During the packing, the cells are assumed to have solid disk or solid sphere geometry regardless of the size of the cell terminals.

### B. Ensemble connectivity

**Figure 4:**
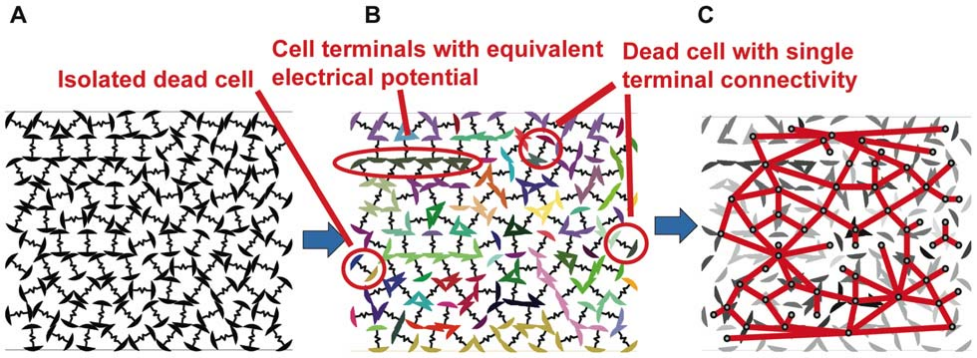
An example cell packing with random cell orientations and the analysis of electrical connectivity. Cell arrangements are constructed and analyzed through a sequence of jammed packing simulations, determination of electrical connectivity, and reduction to a equivalent resistor network: A. the cells are packed with random cell orientation, B. a representation of electrical connectivity, where each color indicates cell terminals of equal electrical potential, and C. the equivalent resistor network for the cell arrangement where the nodes are the points of equivalent electrical potential and the edges are the equivalent resistive connectivity between nodes.

**Figure 5:**
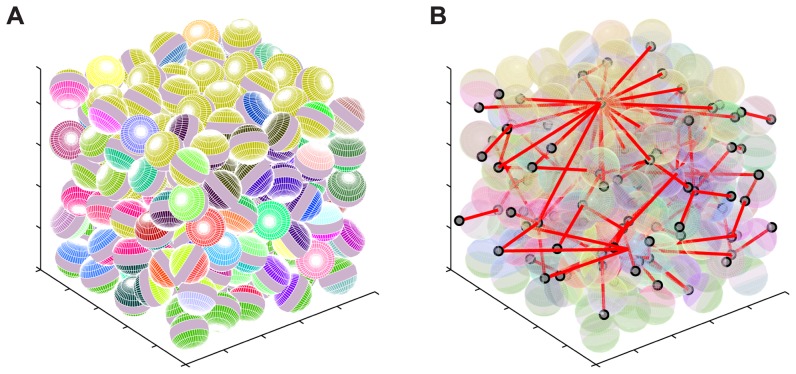
An example three-dimensional cell packing and connectivity.

Figure 4 illustrates the process of simulation and analysis of the connectivity for a 2-dimensional array of cells. First, the cells are placed according the cell packing rules given previously. Subsequently, cell connectivity is determined by analyzing where cell terminals are in contact. The solid disks in the cell packing algorithm are replaced with cells having a gap between the cell terminals. To avoid the current concentration boundary effects discussing in the theory section, we create a “supernode” at the top of the structure connecting all the cell terminals exposed at the top of the structure. A second supernode is created in a similar manner at the bottom of the structure. A node number is then assigned to each group of connected terminals. In Figure 4-A and 4-B, the resistors are only shown for aesthetics. Figure 5-A and 5-B show an indicative three-dimensional packing and the associated electrical connectivity through the structure.

In these simulations, we assume that all cell resistances are equivalent with a value of 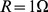 and an ideal current source is connected between the two supernodes. We then compute the voltage difference across each resistor, the current passing through each resistor, and the resulting power dissipated in each resistor.

**Figure 6:**
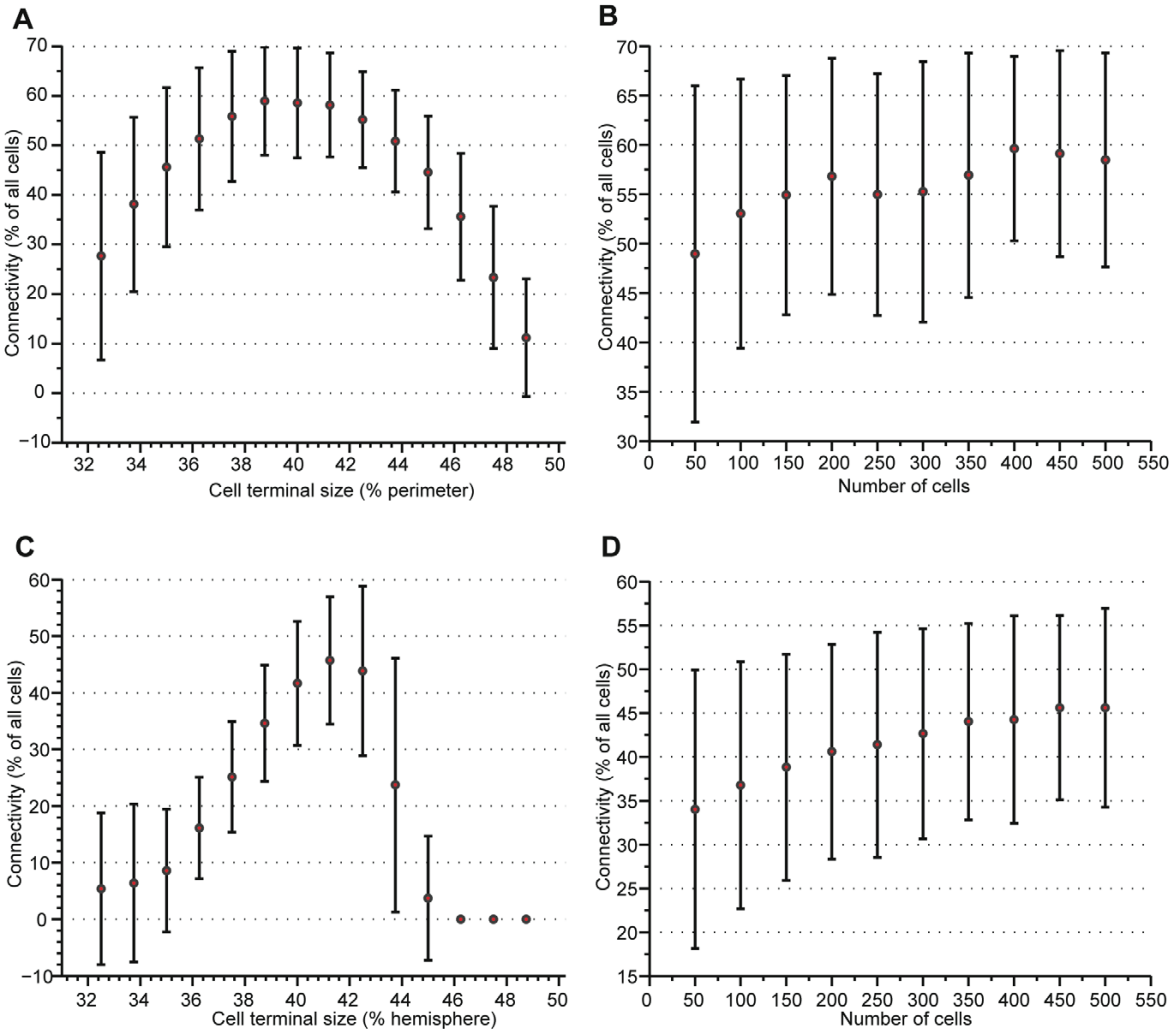
The effects of cell terminal size and cell count on overall connectivity. An analysis of the connectivity of 2- and 3- dimensional cell arrangements as a function of the size of the cell terminals and the number of cells in the arrangement. We conducted 1000 simulations with random packing and random orientation of each cell with the mean and standard deviation shown: A. the percentage of active cells in a 2D packing as a function of the terminal size, B. the percentage of active cells in a 2D packing as a function of the number of cells, C. the percentage of active cells in a 3D packing as a function of the terminal size, and D. the percentage of active cells in a 3D packing as a function of the number of cells. There is a clear optimum cell terminal size for both 2D and 3D cell packings. For small terminal sizes, the lack of connectivity is due to insufficient terminal-to-terminal contact. For large terminal sizes, the lack of connectivity is due to top-to-bottom shorts due to excessive connectivity causing cells to have equivalent potential on both terminals and no current passing through the SMA element. As the number of cells in the structure increases, the connectivity increases logarithmically. In all packings, there are often cells at the boundaries that are not active due to single terminal connectivity. As the number of cells increases, the proportion of boundary cells decreases.

Figure 6 shows the results of the two types of simulations conducted: (1) determining the effect of the cell terminal size on connectivity and uniformity of power dissipation and (2) determining the effect of the size of the cell array on connectivity and uniformity of power dissipation. In each case, we conducted 1000 simulations with random cell orientation to approximate our plan of casting cells in a mold without regard for alignment. Figure 6-A and 6-C, for 2- and 3-dimensional cell packings respectively, show that there is clearly a terminal size for which connectivity is maximized. The optimal terminal size is completely dependent on cell geometry. This analysis should be repeated for alternative cell geometry, e.g. ellipsoids, superballs, etc. From Figure 6-B and 6-D, we conclude that the number of cells in the array is less important than the size of the cell terminals, but that the connectivity increases as the array size increases. This is attributed to the fact that there will be fewer inactive cells near the edges of the array as the size of the array increases.

### C. Momentary disconnects

**Figure 7:**
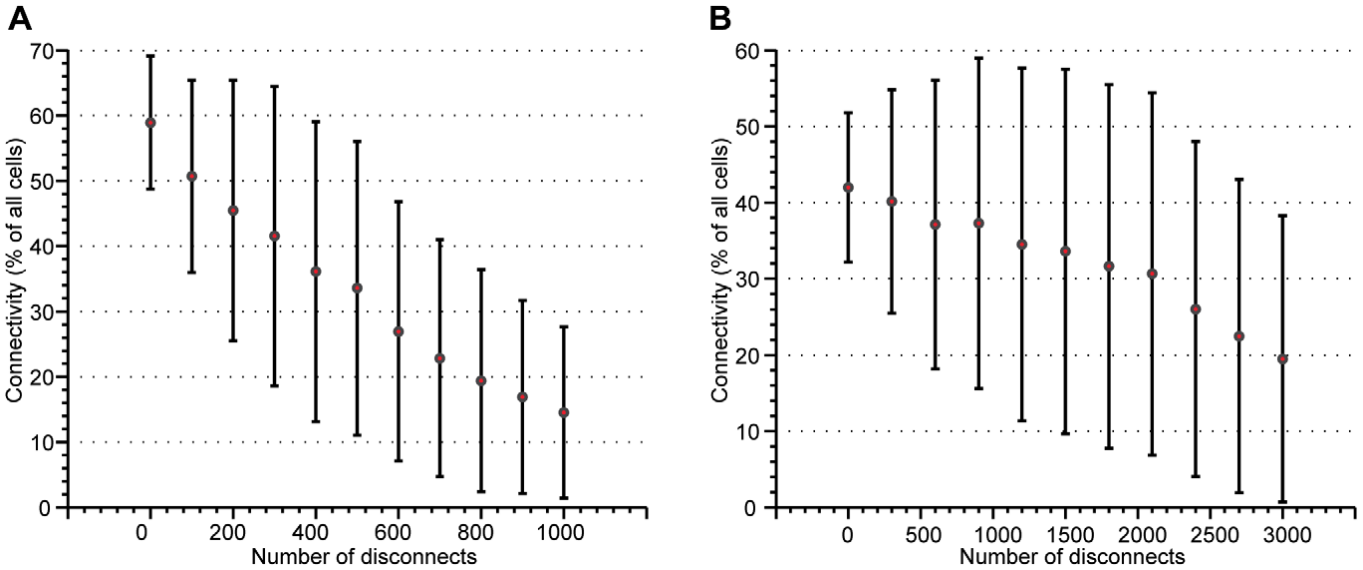
The effects of temporary disconnects on the overall connectivity. It is expected the structure of active cells will have momentary disconnects between adjacent terminals as it is actuated and that cells will fail with age. Here, we analyze the effects of an increasing number of disconnects on the overall connectivity: A. the percentage of active cells in a 2D packing as a function of the number of disconnects and B. the percentage of active cells in a 3D packing as a function of the number of disconnects.

One of the proposed advantages of this cellular approach to active materials is robustness to momentary disconnects and cell failures within the structure. Using binding materials such as soft elastomers, disconnect are expected when the structures is under load and cell failure is inevitable. Figure 7-A and 7-B show the effect of a varying number of disconnects throughout the structure, for 2- and 3- dimensional cell packings respectively. These simulations were conducted using a pad size of 80% of each hemisphere of the cell and 500 packed cells. During each trial, the cells were randomly packed and a random number of cells that would have been in contact were disconnected. The overall percentage of active cells does not decrease considerable from the nominal value given in Figure 6, even when there is a fairly high number of disconnects compared to the total number of internal connections.

## Discussion

In this paper, we have shown that not only can the connectivity of a cell-based structure vary with the design of the cell contacts, but that there exists an optimum design at around 80% of the hemisphere, giving maximum contact with surrounding cells while minimizing shorts in the connectivity graph. In the long term, the cellular method has the potential of introducing a new way of approaching smart materials; an ensemble of small, actuated cells can provide a basis for distributed articulation similar to biological systems.

Though simple conceptually, the cell-based approach described in the paper has several challenges as we move from theory to implementation. The most salient challenge will be to ensure adhesion between cells and the surrounding substrate. This will almost wholly define the shape changing capabilities of the ensemble structure. Another critical challenge will be to balance the stress-strain cycles of the cells with that of the surrounding material to achieve an adequate work cycle for the purposes of the overall structure.

Though the simulations shown herein showed completely random packing and cell orientation (which is the easiest method of implementing the cell-based approach in hardware), overall connectivity of the structure can be increased significantly through even modest amounts of preferential cell alignment (as seen in Figure 2). During the construction of cells, asymmetric mass distribution in the cells and shaking of the mold might be used to achieve preferential alignment, along with a number of other potential methods.

Obvious future work is to begin constructing cells and groups of cells. Work is already under way to construct and evaluate individual cells, a natural precursor to creating two- and three-dimensional groups of cells. After finalizing construction of individual cells with the appropriate controllability, repeatability, and force capabilities, we will complete mechanics-based modeling of the cells and surrounding substrate to compare our modeling results with the physical instantiation. Another interesting theoretical problem is how to determine the internal configuration of the structure through system identification techniques of actuation of the structure and measurement of its deformations.
